# The Effect of COVID-19 Pandemic on Service Sector Sustainability and Growth

**DOI:** 10.3389/fpsyg.2021.633597

**Published:** 2021-05-06

**Authors:** Shihui Xiang, Saad Rasool, Yong Hang, Kamran Javid, Tasawar Javed, Alin Emanuel Artene

**Affiliations:** ^1^Chinese Graduate School, Panyapiwat Institute of Management, Pak Kret, Thailand; ^2^College of Computer and Information Technology Engineering, Hohai University, Nanjing, China; ^3^College of Economics and Management, Jiangsu Maritime Institute, Nanjing, China; ^4^Information and Communication Engineering, Hohai University, Nanjing, China; ^5^Department of Management Science, The Islamia University of Bahawalpur, Bahawalpur, Pakistan; ^6^Faculty of Management in Production and Transportation, Politehnica University of Timisoara, Timiṣoara, Romania; ^7^Research Center in Engineering and Management, Politehnica University of Timisoara, Timisoara, Romania

**Keywords:** COVID-19 pandemic, business sustainability, economic growth, service sector, information technology

## Abstract

Coronavirus disease (COVID-19) is having an unprecedented and unpredictable impact on the world's economy. The pandemic has driven the world toward adapting to the current circumstances regardless of the business, sector, or industry. The coronavirus epidemic (COVID19) has affected the global economy and service sector. The purpose of the current study is to assess the effect of COVID-19 on service sector growth and sustainability. Global sectors and industries are trying to anchor themselves amidst the pandemic. The study focuses on the sectors that are badly hit by the outbreak and discussed the strategies and responses different countries are taking to sustain their economies. This study concludes that the vital role of Information Technology and digitization supports the economies in their fight against the pandemic and helps them sustain themselves amid crises. This study also contributes to the body of literature by suggesting IT-based solutions for various industries to elevate effective responsiveness and avoid significant losses.

## Introduction

Technology has allowed for extensive growth in almost every human interactive field in the last few decades. Information communication technology has allowed the world to become globally connected. This has left a lasting impression on the global market, especially with regards to the trade between developed and developing countries. The global market is within reach for most people, strengthening the economy and individual living standards both. The use of information sharing and communication technology by customers is very significant in today's era of the global market.

The COVID-19 epidemic has hit the world economy very hard, leaving no industry unaffected. In this study, we discuss the gaps in the literature surrounding the economy and economic environments and encourage future research to focus on the stress and well-being explanation, particularly in response to the pandemic decline. Rising protectionism and chauvinism have worsened during this pandemic, accelerating the weakening of the economy. It affects the economy and whole standards and values, altering them to produce an entirely new chain of values. Social distancing and security become more critical than immediacy and good organization. Relocation and shut down of businesses and supply chains are experienced in almost every sector, and even governments have put the economies aside and are hoarding basic necessities. This situation has brought about not only practical social distancing but also economic distancing and has put the world economy in danger.

In such a devastating situation, technology is the only hope to keep the economy on track. Technological instruments are vital resources for the efficient monitoring and control of disease outbreaks since people cannot operate and balance the magnitude and speed of AI devices. Thus, where every country is in the race to develop a COVID-19 vaccine, each country is looking at its technical experts to come forth and play their part in keeping the economy on track. The downfall of the various sectors during this pandemic shows discomforting fallout in which health care is on top. During this pandemic, the healthcare system collapses in many countries, and the overwhelming influx of patients to the hospitals has been disastrous. The global lockdown has reduced supplies to almost zero, and even the mobility of necessities was cut down by more than a quarter. Other than the healthcare system, key industries that are hit harder by lockdown are manufacturing, retail, public services, entertainment, media, the transport industry, and tourism. Information technology plays a vital role in providing accumulative, accurate, and reliable communication. Information technology offers medical and economic benefits of the right to information access; moreover, IT seems to be the only way out during lockdown and social distancing, compensating for economic and business losses. Its economic benefits in such a situation are widespread, including fast communication and novel compensating methods. AI-assisted virtual assistants, chatbots, and information centers help the health sector diagnose and test for COVID-19 and play a vital role in facilitating the maintenance of social distancing to stop the spread. The cryptocurrency trade significantly influenced environmental sustainability, which is econometrically confirmed by the vector error correction model (Mohsin et al., 2020a). The banking sector is symmetrically and asymmetrically affected by the volatility of market risk. Simultaneously, interest rates and exchange rates show minor participation in the fluctuation of banks' returns (Mohsin et al., 2020b). The pandemic outbreak influenced the daily activities of life, and the stock market is negatively affected by it because of the alteration of investor psychology (Naseem et al., 2021).

This study aims to investigate the economic effect of COVID 19 on service sector firms. During this global pandemic, the economic losses are so devastating that global economies are looking at a future recession. Unemployment and poverty are at an all-time low, and IT help is needed to eradicate the catastrophic consequences. This study also established what sectors are badly affected during this pandemic and need thorough rehabilitation. Only a few sectors show fewer losses or are doing well during the current pandemic. The vital question is how information technology can help people and businesses cope with worsening situations worldwide. The study objective is to highlight the impact of COVID-19 on various sectors while determining the worst-hit industry. The current study also emphasizes the role of information technology during COVID-19.

In such a situation, technology is the first and foremost foundation upon which economies are dependent and mitigates the impact of COVID-19. Information communication technology in this digital world plays a key role in combating the pandemic. Technological areas like artificial intelligence, cloud computing, and data science are evidenced to be the only players that have kept working at their full pace during this emergency. The COVID-19 pandemic has hit almost every sector of the economy in every corner of the world. So, these technologies are implemented when allocating resources, treating patients, preventing the spread of the virus, tracking carriers, and monitoring the daily situation of a pandemic, and they are also implemented by various economies to support their businesses in tackling their problems and social activities during the lockdown. Digital technology seems to support companies with IT infrastructures, duly operating online and offline.

In contrast, businesses that are lagging in incorporating IT technology have faced many problems. Social distancing and stay-home restrictions have meant offline businesses suffer the most. However, many businesses that were not operating online before the pandemic have somehow managed to shift their businesses online to fight against the pandemic's economic disaster. Schools and other offline businesses were put on lockdown to prevent the spread of the virus, and many continue to operate though online. This also brings a surge in the demand for online learning applications and work from home office solutions.

Moreover, there are two sectors that have been affected the most during this pandemic: health and tourism. Many countries' healthcare systems have been partly or entirely interrupted. Besides the high-tech climate, the healthcare industry has taken a devastating hit due to the multiplication of COVID 19 patients and the lack of healthcare staff, keeping people with routine health problems out of hospitals. This sector actively moved toward telemedicine telehealth solutions. This move seemed to be very effective in ensuring routine patients stay home, allocating the hospital's resources toward designated COVID-19 wards. The abrupt and strict lockdown in the urban and rural areas has halted traffic, and, as a result, there has been a global decrease in the influx of road-related emergency patients. Patient-engaged video surveillance and robotic intervention to deliver medicine right to the patients' beds has kept the healthcare personnel safe from contracting the virus.

On the other hand, the badly thumped sector was tourism. Restriction on public gatherings and suspension of flight operations throughout the world has had a negative impact on this industry. Tourism and allied services have also received the same level of stress. Many businesses located in tourism hot spots have had to shut down to keep people away from the tourist spots.

Both sectors show a mix of both extremes of having supportive IT infrastructure and not having IT or online infrastructure. This study will explore the impact of COVID-19 on both types of businesses to analyze how an IT infrastructure helps businesses to maintain their economic activities during such situations. We intend to answer the following questions:

- How has COVID-19 affected firms in the service sector, and how has an industry IT infrastructure made them capable of fighting COVID-19 in a lockdown situation?
- How does an IT infrastructure play a vital role in keeping businesses running and revamping them after strict lockdown?
- How does IT infrastructure play an essential role in ensuring that companies are operational and refitted after strict lockdown?
- How did different countries respond to COVID-19 to continue their economic operation, and what is their recommendation regarding IT integration?

According to the International Labor Organization (ILO), the impact of COVID-19 varies depending on the specific social and economic sectors. This study is based on the ILO's assessment of the sectoral effects and captured industries. This study will establish that it is the weakness of various companies' IT infrastructures that causes the economies to collapse. Public health concerns are forcing companies to work from home, and these companies are facing a massive loss of opportunity. Many manufacturers import their required raw material and spare parts from China, which is currently under lockdown. Manufacturing processes have had to shut down as a result. There was only one public health sector working with its full strength and workforce during this pandemic; all industries were working either partially or not. Secondly, the industry hit the worst have been tourism and its allied sectors, such as aviation and the public transport sector. The research aims to examine the effect on major industries that contribute to the economy, emphasizing their IT infrastructure.

## Literature Review

### Use of IT in the Media and Entertainment Industry

The recent lockdown highlighted the importance of the media and entertainment sector, as it plays a vital role in elevating public morale. During a pandemic, people have been instructed to stay at home, and people have thus turned toward entertainment, education, and cultural activities, which has, in turn, overloaded streaming and other online services. Simultaneously, it has been tough for production houses to keep their activities running, as recording shows and live broadcasting has become more challenging due to social distancing. Due to the pandemic, this sector has also faced unemployment and clogged production. Job losses and the fall in economic returns have been exacerbated by the increased volatility of media and culture during the pandemic. Due to the media industry's shutdown, the cost of the pandemic in terms of lost media revenue has been approximately USD10 billion (Hall, 2020).

During the strict social distancing and lockdown situation, 79.7% of the workforce of the arts, recreation, and entertainment industry lost their jobs or were placed on forced leave in the United Kingdom (Mangan, 2020). Similarly, in the Philippines, the entertainment workforce fell by 55% in April 2020 (Authority, 2018). The same is witnessed in Australia, where performing arts and other cultural activities fell by 29.5% in March and April (Statistics, 2020). Moreover, significant scaling of businesses was done, and the impact this has had is visible in both live and recorded transmission. The media industry workforce was shifted to other sectors to ensure employment and provision of services in public health; in Georgia, for example, film set workers were moved to help build hospitals.

A similar situation is observed in the media industries (news agencies, motion pictures, and broadcasting) of Brazil and the United States. According to the Institute of Brasileiro de Geografia e Estatistica (IBGE), the audiovisual news agency and editing industry production fell by around 14.8% in March 2020. According to the United States Bureau of Labor Statistics in Current Employment Statistics survey, the motion picture sector fell by 52%. The broadcasting sector fell by 9% during this lockdown in May 2020.

In the media and entertainment industry, various sectors like live broadcasting and recorded production used IT to capitalize on themselves and technology to monetize their productions. However, live media broadcasting could not take much advantage of IT to conserve their quality association with their audience. Still, recorded programs that use stipulated music and other audiovisual content showed much improvement in terms of streaming and its associated IT infrastructure. It is also observed that during the pandemic, the IT infrastructure is pacing the productions. Hall (2020) indicated that various online platforms and applications play their role in bringing up creative content with a copyrights issue.

Technological and IT infrastructure also helps filmmakers maintain social distancing. An example of this is by replacing the extras with robots and AR visual images, such as in France 24, 2020: “Robot helps Turkish sitcom keep cameras rolling in the age of COVID-19.” Maintaining social distance between actors has also meant the provision of equipment that allows actors to film themselves. Similarly, those working with voiceover and dubbing are provided with the equipment to record themselves or are sent to rent cars with equipment onboard near their houses so they can work remotely. This is how the COVID-19 pandemic has hit the media and cultural industry. Information technology inclusion in this industry has allowed the industry to hit the ground running, but it would not have been easy without sufficient technology and IT services. Therefore, it is proposed that IT service deployment is often a valuable tool to integrate into existing systems. Flim Directors are using the robot camera for making a new video.

### COVID-19 and Public Service

After World War II, the COVID-19 pandemic is largely considered the worst crisis the world has in recent history. It has had a severe impact on healthcare systems around the world, the global economy, and society as a whole. Much like in response to combat, the nations of the world, on the call of the WHO and UN Secretary General, have moved rapidly toward fighting this pandemic, and the public service has been placed as the vanguard in this situation. Public service departments maintaining social distancing to keep the public safety have limited their operations. However, they also provide public services through IT infrastructure both online and remotely. COVID-19 is the first epidemic in human history in which technology and social media are used to shield people while being physically isolated. The departments that have been impacted in public services are transportation, the health industry, the media industry, etc. Some cultural events, airports, and other services that involve large public gatherings are suspended till further notice. In such settings, the IT infrastructure is the only solution to provide updated information, surveillance, and jobs that are currently impossible to execute. Examples of this include working remotely from home and tracking people's movements to contain the virus.

Along with health and education experts, public servants also help prevent virus spread and aid recovery. During this pandemic, many public servants have worked from home using the technology their respective departments have. The public servants were also part of the prevention of COVID-19, and technology has helped them do their daily office work to reduce social contacts by providing essential services using only technology. However, in some countries, not all the departments have been using technology and had very little to no IT infrastructure. In this case, they deploy the already available online platforms or cut down their operations to reduce person-to-person contact. Countries with high-tech environments have shifted their education, health, and public services to be online and have thought of ways to ensure e-governments can maintain their technical capacity. Information technology is capable of facilitating both health and social concerns.

### COVID-19 and Food Retail

The drastic coronavirus pandemic has caused spikes in demand for certain goods because of panic buying. The demands for other goods have decreased frantically because of the decline in use. Nielsen, the British Retail Consortium, and the Commonwealth Bank of Australia mentioned that the surge in demand for essential products like rice and wheat, toilet paper, and various other food items has been much higher than the last year (Sarfraz et al., 2018, 2020a; ABC News, 2020; Briefs and Books, 2020; Lobach, 2020; Saria and Raheja, 2020; Bentall et al., 2021).

Pak (2020) also mentioned in his studies that “As China recovers from COVID-19, small businesses are struggling to re-open,” and medium-sized enterprises will face the hardest hit, as they have no alternate arrangements like e-commerce and IT infrastructure. These businesses often lack safety nets when workers become sick. IT infrastructure and associated technologies are used to tackle the paramount pandemic hygiene and prevention measures. These include self-checkouts to decrease contact between workers, reducing cash purchases, adding protectors to counters, and re-stocking shelves using schedules. There is a change of −0.9% employment in the Australian accommodation and food services sector from March 2019 to March 2020 (Vandenbroek, 2020).

### COVID-19 and the Transport Industry

The people's movement is changing with the help of technology and innovation. However, the coronavirus pandemic is likely to put moving wheels to a grinding halt. Technology and innovation can accelerate transport instead of impeding it, especially in terms of digitizing transport in urban areas. In some literature, urban transportation is the best way to embrace innovative technologies and IT infrastructure. In her blog about the transport economic situation in COVID-19, Tatiana Peralta Quiros mentioned that it is important to reopen the transport industry, keeping public safety in mind. For this, they are analyzing the moving patterns of the people to know where and how people are moving at the local and national levels. The data from the existing and new IT applications must be keenly observed to update the people in real time about the traffic and congestion on the roads, malls, parks, etc. Digital technology must be used to track the virus's spread as the situation is frenzied, unprecedented, and keeps changing every day. Covid-19 impact on air travel has been overwhelming (Gössling et al., 2020).

During this coronavirus pandemic, the burden on organizations has changed from moving people to maintaining a core transportation system functional with a skeleton crew to guarantee the continuing movement of freight and critical employees. In pre- COVID-19 times, technology and IT was never used in transport. Wayfinding through navigators is a comparatively old concept, but using it to keep social distancing will be an innovative idea. Installation of the outdoor kiosk to tackle crowded situations is also in place, and many of these have implemented touch-less screens. People's movement surveillance can limit the spread of COVID-19, but it may raise privacy issues (Hutchinson, 2020).

### COVID-19 and the Healthcare Sector

Given that the healthcare sector provides spiritual services to the public, its value is visible in its demonstrable ability to improve both the economy and job ratings. The healthcare sector always comes first after an emergency and workers. The health sector is the first to fight an emergency. Digitalization and technology are becoming a vital part of the health sector. Technological advancements like online doctor consultations, telemedicine, and other pharmaceutical and treatment-related applications play an important role in providing the best health services (Ungureanu et al., 2019; Sarfraz et al., 2020b,c; World Health Organization, 2020).

In the context of the recent COVID-19 spread, artificial intelligence and related IT infrastructure can help health experts in many ways. In some regions, mobile applications were introduced to track the COVID-19 situation and collect the location-based data of people suspected to be infected. Also, call centers were established with medical experts to help patients with severe symptoms. Information technology also played an essential role in providing the updated information, training the health experts, and guiding them according to the situation. This can also help monitor service quality, management, and transparency in remote areas (Salsberg and Martiniano, 2018; Keesara et al., 2020).

The health sector is the sector most impacted by the pandemic; healthcare personnel are short of PPE and have seen cases of infection. The has been a shortage of both beds and other equipment, including ventilators. Technological advancement plays a role in flattening the spike of the confirmed cases and reducing the flux of the patients toward hospitals (Smith et al., 2020; Wang et al., 2020).

### COVID-19 and the Education Sector

The spread of COVID-19 was witnessed throughout the world in April. In March 2020, the WHO had already declared it a global emergency. In April 2020, most countries shut down their schools, colleges, and universities, keeping public safety in mind. The purpose of the closure of educational institutes was to slow down the spread of covid-19 by keeping people in their houses and away from gatherings. This encouraged distance and online learning using technology, as 91.4% of the students, around 63 million teachers, and other education-related staff were severely impacted. To tackle the raised educational challenges in school closures, technology was embraced by schools, and it has shown its value in providing virtual learning-based platforms that can mitigate the loss of education. Teachers and students show adaptability in using technology-based platforms for online learning as an alternative to the existing conventional classroom system (Chick et al., 2020; Ting et al., 2020).

Technology also helps sustain teachers' salaries and other personal training by the use of online instruments and media as people transfer their teaching process to remote learning. While transforming the educational system into a completely new digitalization environment and technology-based schooling, the teachers need strong digital and technical skills. Other ongoing studies in many countries yield insight into how online and distant learning techniques using digitization and technology can help developed and developing countries manage their responsive measures during the pandemic situation (Park et al., 2020; Verawardina et al., 2020).

### COVID-19 and the Tourism Industry

Within 6 months of the day COVID-19 was initially announced in China, the spread of coronavirus was declared a global pandemic. It has had an unprecedented impact on almost everything, including activity and movement. It has left mammoth adverse effects on social, political, and economic sectors, being irreversible in some sectors. The ripples of the COVID-19 keep threatening people's health and life. Global and domestic governments have imposed different levels and sanctions on their citizens, including travel bans, restrict gatherings, stay-home orders, self-quarantine, and other business closures and time-specific restrains. The countries whose economies are hospitality-based have felt a significant negative impact on their economies as travel, tourism, and services such as aviation have come to a halt.

The hardest-hit sector of all is travel and tourism because of partial and full lockdown situations worldwide. The travel and tourism industry and other associated sectors have fallen to a small fraction compared to what they contributed in pre-COVID-19 times. Where other sectors like health, education, public transport, media, and hospitality are starting to reopen following SOPs of COVID-19, the travel and tourism sector is still at a halt due to its fragility to the whims of the virus. Moreover, other sectors transform their operations to online platforms to various degrees because of nature and adaptability. This sector is facing challenges in adopting the electronic form of tourism: the lack of IT infrastructure and technological advancement and limitation of implementing technology-based tourism. In this regard travel and tourism sector behaves differently by nature. Crises in the tourism sector are not new by any means, but from an economic point of view, COVID-19 has a more devastating impact than previous crises in recent history (Gossling, Scott, and Michael). Like all the historical crises, this pandemic also brings irreversible damage to this industry. Despite being under the discussion of researchers, e-tourism is still not implemented to an extent where it can show resilience and fight against such pandemics. Information technology and tourism have always had a challenging relationship (Werthner and Klein, 1999).

In the future post-COVID-19 era, tourism is not expected to be the same as it was in pre-COVID-19 times. The tourism sector's situation needs to be tactically dealt with to recuperate the socio-economic stability after the COVID-19. The tourism industry's subsectors have also taken bad hits, including industries of aviation, road transportation, recreation, accommodation, and the food supply chain. The tourism industry's digitalization also proposes the changes in these subsectors at a massive level. Innovative and technology-based solutions are proposed concerning travelers' safety and hygiene. The modern socio-economic system is based on the hospitality and tourism industry, as many countries are shifting their economies from natural resources to tourism. When the crises intimately affect this industry, it can even turn into a devastating slump. The tourism industry faces different natures than other sectors, as IT cannot do much about it except speeding up the booking, destination hunting, and destination recommendation process, etc. To sustain the tourism industry economy in the post-COVID-19 times, tourism brands have had to adopt innovative and bold IT infrastructure and use broader technology scales. Technological and digital innovations are the future of tourism (Huang et al., 2016; Rao and Krantz, 2020a). Chat-bots, online bookings, and journey planners are already in place. Still, this sector is now looking forward to implementing virtual tourism, robot-based services, emotion monitoring, and the internet of everything technologies. This will define tourism in a completely different way. Many hotels already implement robots to provide hotel and housekeeping services to mitigate the human-to-human interaction (Afsarmanesh and Camarinha-Matos, 2000; Palmer and McCole, 2000).

Many researchers are putting forward creative ideas to reduce humans interaction in the tourism and travel industry to minimize losses. The refined keywords from the literature can address the study's objective more precisely. New standards of room cleaning, elevator operations, locks, switches, and other tactile objects will be regulated and reworked. Some researchers work on contact-free receptions and customer voice-operated complaint and suggestion books. Other IT techniques like artificial intelligence, deep learning, and machine learning will predict customer needs, mobility patterns, and behaviors. Recreational places where social distancing cannot be implemented effectively and the places that host many people will also be digitalized using augmented and virtual reality tours (Chirisa et al., 2020; Kwok and Koh, 2020).

Some researchers proposed VR techniques for unique travel experiences in the same scenario. The movement restrictions on the frequent travelers and tourists confined them to their homes; traveling is not currently an option now, and it is unknown if and when life will return to normal. The travelers will soon find the VR apps for their choice of destinations. VR-based traveling apps are as helpful in ordinary life as in covid-19 times. The travelers can check out their destination in 3D even before they arrive to avoid the hassle of airport requirements, tickets, and holiday arrangements (Bhuiyan et al., 2020; Rao and Krantz, 2020b; Sigala, 2020).

**Figure 1:**
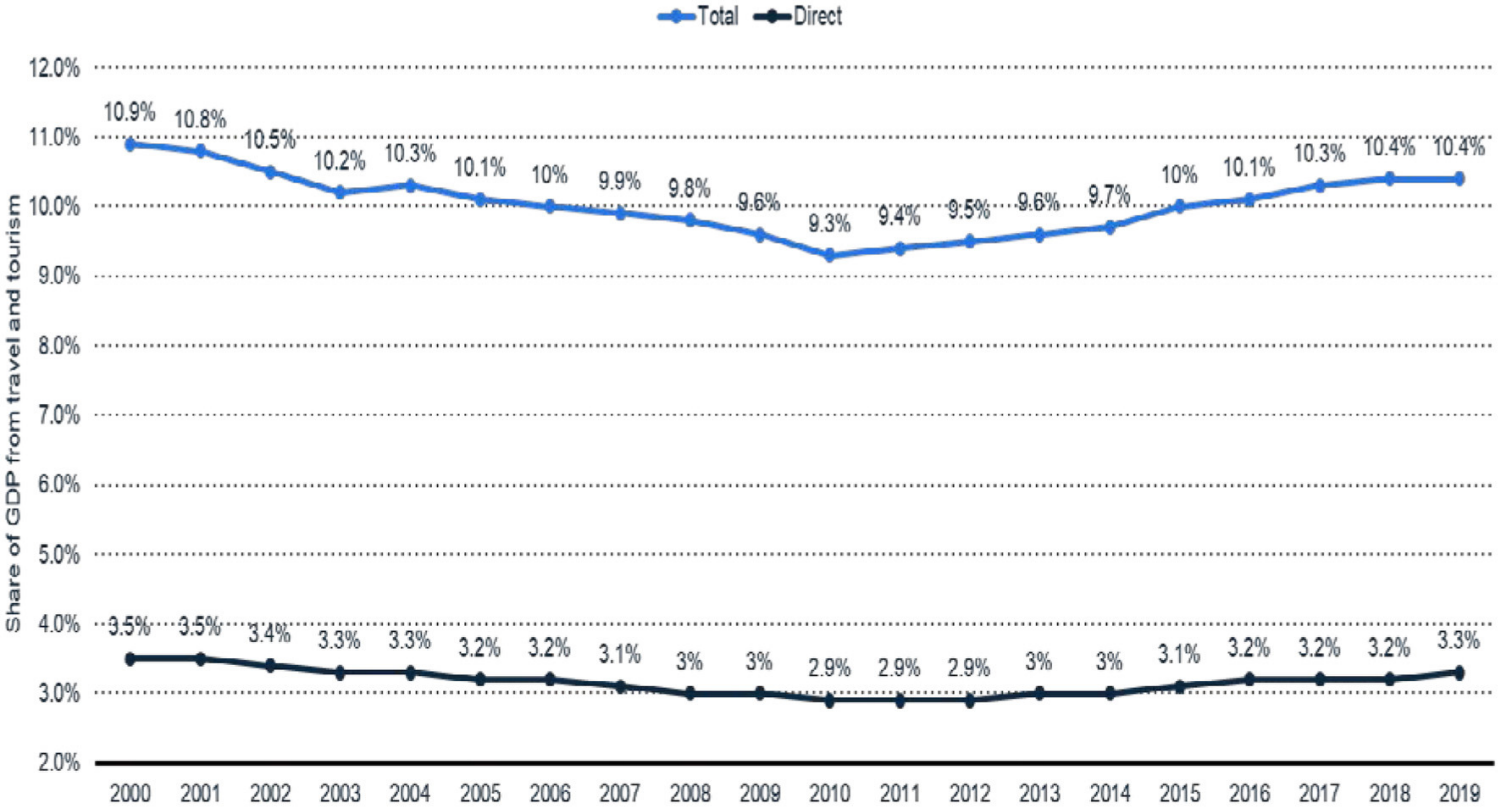
Worldwide GDP share (travel and tourism industry). Data Source: (Statista, 2020a).

Figure 1 shows the worldwide GDP share of the travel and tourism industry from 2000 to 2019. Travel and tourism made a 3.3% direct contribution to the global gross domestic product in 2019, but it was stable at 3.2% from 2016 to 2018. The direct contribution to the travel and tourism industry's global gross domestic product was 10.9% in 2000 and 10.4% in 2019. The direct contribution refers to internal spending on travel and tourism.

**Figure 2:**
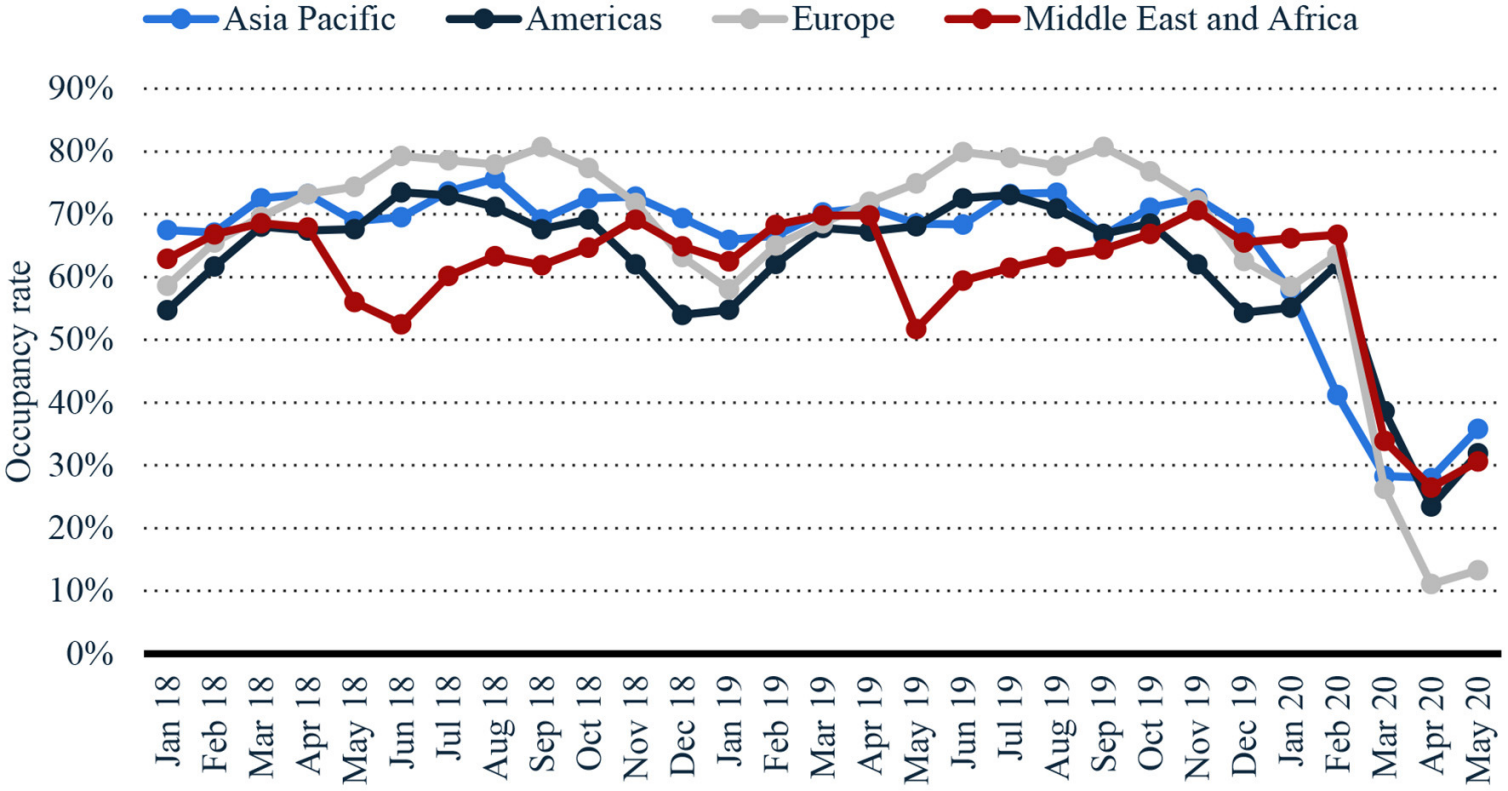
Worldwide Monthly hotel occupancy rates (2018–2020). Data Source: (Statista, 2020b).

Figure 2 presents the monthly hotel occupancy rate from Jan 2018 to May 2020. The impact of COVID-19 can be seen on the hotel occupancy. The results show that hotel occupancy has been dropped significantly due to travel restrictions. Europe hotel occupancy was declined by 82.3% in May 2020. Indonesia has faced the highest decline in tourist arrivals than other Asian Pacific nations.

**Figure 3:**
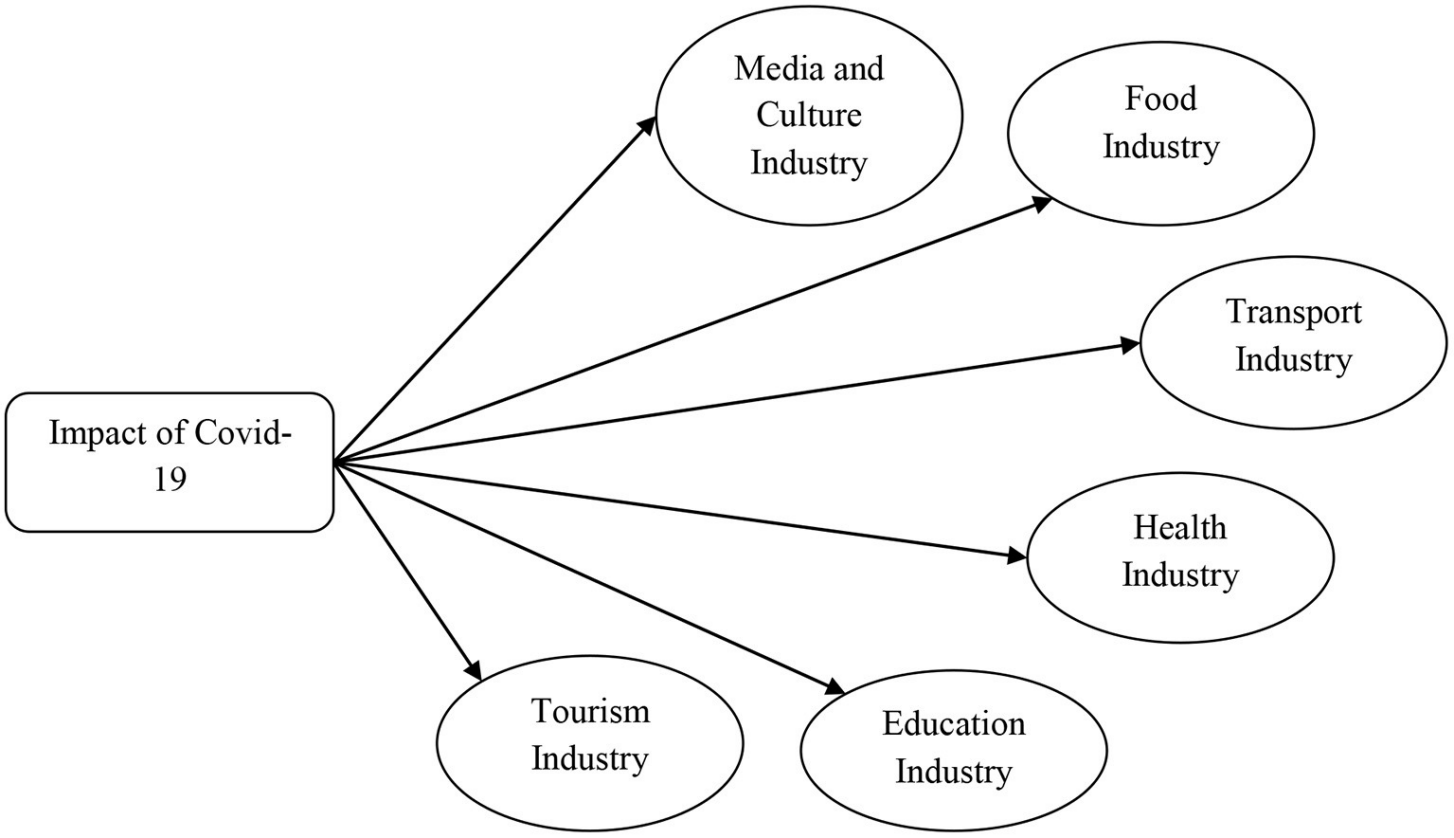
Framework of the study.

Figure 3 shows the impact of COVID-19 on the different service sector industries.

## Research Methodology

The current study was carried out during the pandemic period and at the height of COVID-19 with a strict worldwide lockdown situation. Therefore, the researchers used publicly accessible data to analyze the ongoing global recession based on national website data. The study's goal is to evaluate the adverse effects of COVID-19 on various industries, such as education, health, public services, and tourism. For evaluation purposes, the researcher performed the literature review, including reviews of books, journal articles, official websites, and encyclopedias that recorded the data for information dissemination (Hoque et al., 2020). These sources are considered secondary data utilized for analysis published in academic journals, historical records, government documents, and statistical databases. The current research focuses on keywords in the literature to conclude and establish the recommendations. We used a keyword search to address the study's objective and the concepts we found in the literature. The keywords are more refined for entire synonyms and acronyms from essential literature that can address the study's objective (Hoque et al., 2020).

## Results and Discussion

This section of the study entails analyzing and discussing previous literature. The literature shows that COVID-19 has significantly influenced all industries, including tourism, which is considered the worst-hit industry. The seriousness of COVID-19 increased in China significantly, and it has been observed that unrest is observed across the globe. The turbulence has increased rapidly due to fear of people exiting from their houses, and nearly all flights have been canceled worldwide. People have not been allowed and willing to leave their homes and go outside, and countries have placed restrictions on movement that affect associated industries. The tourism industry is the worst hit in China, as it depends upon people flying to China from the outside world. The current pandemic era has negatively affected the business sector. As stated in articles, the logistics, hotel, and airline industries have been severely damaged due to corona spread. Thus, it has been known that coronavirus has killed millions of people around the world and has devastated the tourism industry. It is predicted that the situation will last longer, as it has suspended outbound tourism. People all around the world are observed to be in terror of the coronavirus due to its severity and rapid spread. The whole environment has become infectious and dangerous for people. The social intermingling of people or social gatherings at public places contributes to the disease's large-scale spread. It has been observed that people worldwide have reduced or eliminated their relationships and contacts with the Chinese population and their businesses. Furthermore, people have reduced travel to China for tourism purposes, resulting in a substantial economic downturn.

On the other hand, Chinese people are prohibited from traveling outside the country due to the virus's harmful spread. Due to the current movement control situation, the whole population is stuck, and the hospitality industry has received the worst impact worldwide. The hotels and restaurants are empty due to the absence of customers. The transportation industry has also experienced a significant loss due to a lack of passengers. The overall economy has been negatively affected due to the emergence of COVID-19 globally, as the situation has become a worldwide pandemic (Hoque et al., 2020).

Previous literature analysis also shows that the emergence of the coronavirus has affected the tourism industry significantly and has become severe over time, and people have become afraid of each other. Hence, the government announced the lockdown and movement control to prevent the spread. Historical reports have identified that immediate contact with humans during December 2019 transmitted the virus locally, which caused a large spread. The virus has affected regular human life as the rate of infection has risen and it is locally transmitted. In China, people are in fear and avoid communication and business relationships with Chinese companies. The travel industry was also impacted due to the Chinese government's lockdown situation meant to prohibit the coronavirus spread. The coronavirus is considered a significant threat to the tourism industry since it has slowed down the economy and activity in various relevant sectors (Pastor, 2020).

There is no doubt that the futures of many businesses are uncertain now, as almost one-third of the world's businesses have suspended their operations. Businesses need financial stability to keep themselves up and running in the post-COVID-19 scenario. Similarly, the media industry is looking for sustainability and financial support to enable;e them to serve the world news, entertainment, and information. Along with this, amid the COVID-19 pandemic, the IT infrastructure supports the media industry. However, the pandemic has had such severe consequences that it is still not enough for their survival. More equipment and innovative IT adoption are required in the sector to run their operations. Information and communication techniques for validating news, creating content, and broadcasting still lack an IT infrastructure.

In the same way, the food industry, transport industry, health sector, education sector, and tourism still need to significantly innovate their IT infrastructures. All of the sectors and industries listed must involve digitalization and technology to respond to crises appropriately. Technology has been playing a prominent role in helping and maintaining frontier jobs. Technology has also reached the debatable stage; the pandemic has also defied the industrial and societal IT structure. The health sector's IT infrastructure is much more sophisticated than any other industry, but this sector is the most challenged one in this pandemic. There is thus still space for improvement, which indicates the gap in research and the consequent presence of opportunity. The decline of the restaurants and food industry during this pandemic also prompt the stakeholders to adopt the technology and digitization to respond more effectively. Likewise, the tourism industry is the most suppressed during this pandemic. It has to change significantly; researchers and engineers have been pitching their innovative ideas to be implemented in the tourism industry to sustain it both amid the pandemic and in post-COVID-19 times.

Amid the COVID-19 crises, many different countries use various technologies to support their businesses and economies and contain the virus. For monitoring the people, the Israeli government allowed their agencies to record and track their citizen's location for 30 days. South Korea, China, and Taiwan also use location-tracking services to limit virus transmission. Defiance of lockdown in different countries was also addressed using location-tracking technology, such as in Germany and Italy, where the user's data integrity laws are quite strict, and the technology is used to control the public gatherings and keep track of social distancing. The United Kingdom also uses mobile application technology for self-reporting of the symptoms. India and Pakistan launched similar applications that allow people to self-report the cases and danger and safe zone, and there is also an option for if someone might have met somebody who then later tested positive for COVID-19. Smart imaging, drones, and robotics are also used in China, Singapore, and Italy for various services.

## Discussion and Recommendations

A country such as China has traditionally collected huge revenue from foreign tourism, ~127.3 billion USD, but the number of tourists has been drastically reduced. Moreover, flights have been canceled and restricted put in place for travel to and from China from other parts of the world. Hence, tourist activities have decreased rapidly. Reports have been published on the loss that occurred in various industries due to COVID-19. It is reported that a similar situation will carry on, and the situation is expected to last for the next few months unless the complete remedy does not play its role. COVID-19 has affected several service industries such as tourism and hospitality, transportation, and restaurants. The government has have imposed a lockdown to avoid the significant spread of the virus. Due to considerable losses to multiple industries, worldwide, business operators have encouraged business operators to act and develop counter-strategies for businesses' survival by taking extra measures that have not been fruitful in avoiding or reducing the virus's spread. However, the tourism industry is currently at a standstill position in the face of these difficulties. The reduction in tourism has led to the loss of business for various industries and resulted in huge losses in these areas. Similarly, the situation has been observed, and we reported that the education industry, transportation industry, hotel, and restaurant industry have faced the worst economic hit due to COVID-19. The practitioners have suggested using information technology to avoid the threat.

The COVID-19 crises necessitate significant changes to the media and entertainment industry's conventional work industry. The potential solution for the media and entertainment industry to sustain the business and avoid economic collapse might not be good news for the free viewers. The local broadcasters have no other choice but to use paywalls on their content by collaborating with Apple, Facebook, and Google for revenue sharing. Content monetization can be an excellent solution to sustain the business and attract benevolent donors. Meanwhile, deep learning and big data techniques can be monitored and useful in content automation.

Maintaining social distance levels is in line with the limitations placed on people's movement, and mass transit is strongly discouraged. Even after the COVID-19 pandemic, digital transformation could be the future of transport. Alongside the automated transport of cars, AI is also likely to be applied in a certain way in this sector using predictive transport analytical techniques. Autonomous distribution and dispatching operations with AI techniques are recommended to improve preventive steps.

COVID-19 affects the education sectors beyond the learning of students. The schools' closure put this industry at significant risk of unemployment and, at worst, complete school closure. To keep the education industry playing its role in educating the people and contributing to the world economy and employment, it is mandatory to revolutionize the education sector. Education revolution 4.0 is already equipping the education sector with technology and innovative learning ideas. The recent pandemic has provided a new direction to be included in the Education 4.0 scenario. Long-distance learning is not a new idea, but its vitality is acknowledged in this pandemic. Many solutions were put forth by the researchers and educationists. Global libraries, interactive learning applications, and other tools to support students, teachers, and parents are the future of the education sector, and these have to be seriously considered. Bloom and Book share are some very innovative solutions recently introduced to stay-at-home learning.

There is no doubt that the COVID-19 lockdown has ground the restaurants and hotel industries to a halt. These industries have equally felt the impact of COVID-19. Despite the difficulties facing this sector, the online ordering service supports the company to a degree. Like other industries, the food industry has also faced unprecedented conditions. The lack of IT infrastructures and the restricted use of technology has proven to be critical threats to the industry. However, this condition also leads researchers to digitize and innovate the food industry. The reinvention of the food sector based on the data mining techniques to know the customer needs can enhance trust. In a new environment where the customer is restricted from moving around and dining in the restaurants, the marketers must revolutionize themselves based on the IT infrastructure, such as through a robotic waiter service, AI, and CCTV to monitor the crowd, reserving the tables remotely can convert this challenge into an opportunity.

The tourism industry is working hard to plan for its recovery after the pandemic because, in the last 10 months, the tourism industry is on the verge of collapse. Tourism-based economies face the biggest challenge in their history. The efforts are placed toward the recovery of the tourism industry, keeping the strategies for social distancing and the tourists' safety. As discussed earlier, virtual and augmented reality are immersive solutions for the tourism industry. Only VR and AR can accelerate the travel and tourism industry's comeback. These technologies not only offer the solutions to overcome the outbreak challenge but also retain the enticement of tourists to travel in a virtual environment without being a victim of the virus and can act as the tentative getaway for the tourists who are not willing to travel unless the outbreak has decreased to a minimal level.

## Conclusion

The education industry, health sector, public services, and tourism industry have faced the worst financial setbacks due to the present coronavirus. The airline industry, both at the international and domestic level, has been shut down, as most flights have been canceled due to insufficient passengers and movement control orders worldwide in different countries. The number of tourists and passengers has been rapidly reduced due to panic and the spread of disease. Companies are facing vital challenges around the world. Information technology can play a pivotal role in the firms' sustainability and growth. The government should support small–medium enterprises.

## Author Contributions

All authors listed have made a substantial, direct and intellectual contribution to the work, and approved it for publication.

## Conflict of Interest

The authors declare that the research was conducted in the absence of any commercial or financial relationships that could be construed as a potential conflict of interest.
